# Supplementary material for: Predicting visual function by interpreting a neuronal wiring diagram
Source: Nature. 2024 Oct 2;634(8032):113–23. doi: 10.1038/s41586-024-07953-5 (PMC11446822; doi:10.1038/s41586-024-07953-5)
Supplement: Supplementary file 5 — CRF and ERF predictions for individual Dm3 cells. The CRF is predicted for each target cell by mapping the top five monosynaptic pathways from hexel source types to the target Dm3 type. The ERF is predicted using disynaptic pathways that pass through the top ten intermediary types (ranking from Extended Data Fig. 7). As a set of disynaptic pathways passes from multiple hexel source types through each intermediary to the target, for brevity only one representative starting from a single hexel type is shown, the pathway in the set with maximal strength (red line, Extended Data Fig. 7). The centre of the Tm1–Dm3 CRF (Methods) is indicated by the cyan star, to aid comparison of locations across maps. Next to each map are the maximum and sum over all hexels, in units of 0.01%. In the last panel, all ellipse approximations are shown together for comparison, scaled up by ×3 relative to maps. The dashed ellipse approximates the Tm1–Dm3 CRF, and scale bars indicate one lattice constant and p and q axes. Each file name contains the ID of the target cell in v783. [file 41586_2024_7953_MOESM5_ESM.zip › DataS3/Dm3q/720575940639399502.pdf]

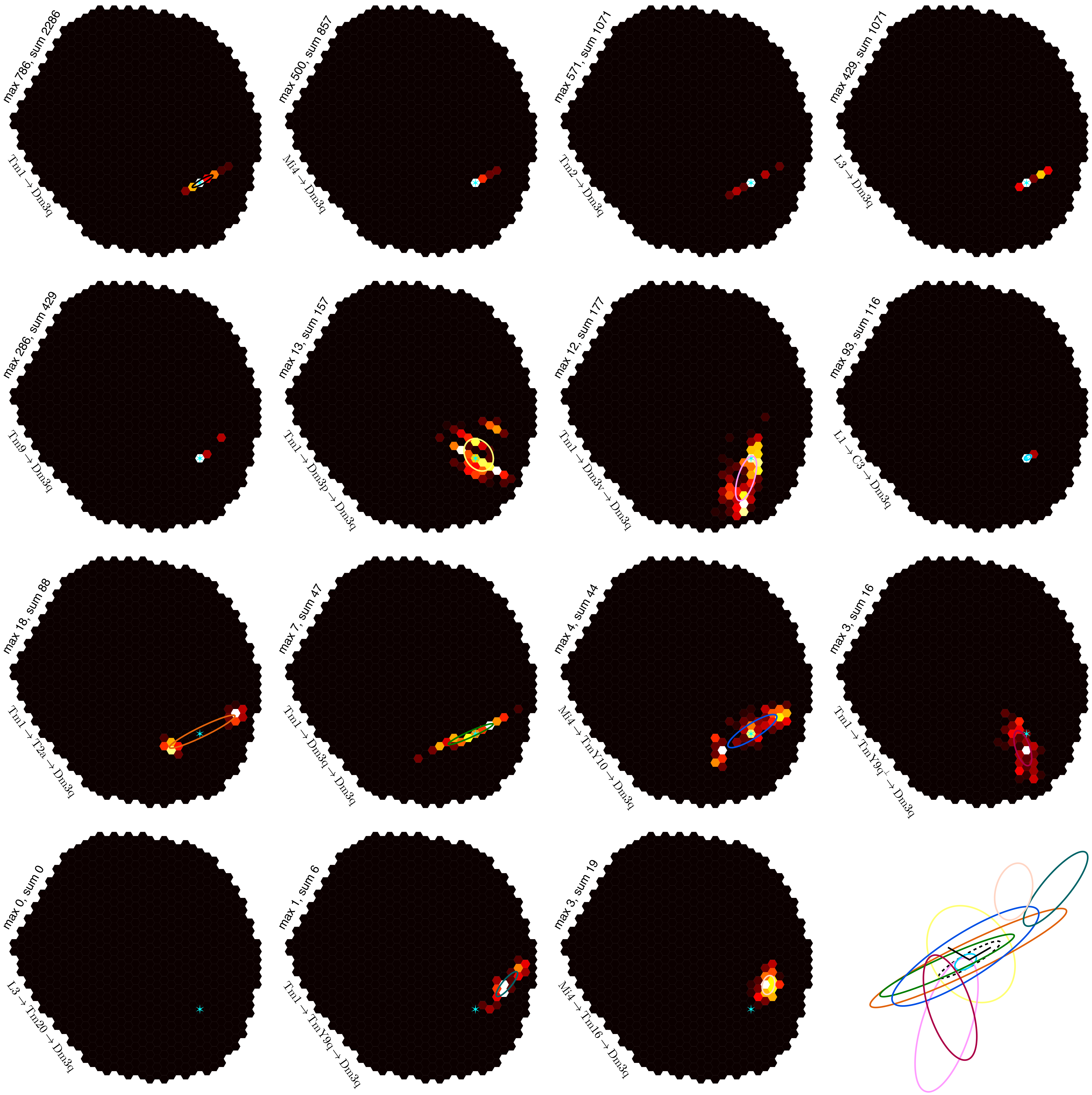
max 786, sum 2286
Tm1 → Dm3q
max 500, sum 857
Mi4 → Dm3q
max 571, sum 1071
Tm2 → Dm3q
max 429, sum 1071
L3 → Dm3q
max 286, sum 429
Tm9 → Dm3q
max 13, sum 157
Tm1 → Dm3p → Dm3q
max 12, sum 177
Tm1 → Dm3v → Dm3q
max 93, sum 116
L1 → C3 → Dm3q
max 18, sum 88
Tm1 → T2a → Dm3q
max 7, sum 47
Tm1 → Dm3q → Dm3q
max 4, sum 44
Mi4 → TmY10 → Dm3q
max 3, sum 16
Tm1 → TmY9q⊥ → Dm3q
max 0, sum 0
L3 → Tm20 → Dm3q
max 1, sum 6
Tm1 → TmY9q → Dm3q
max 3, sum 19
Mi4 → Tm16 → Dm3q
